# Characterization of the Mycobacterial MSMEG-3762/63 Efflux Pump in *Mycobacterium smegmatis* Drug Efflux

**DOI:** 10.3389/fmicb.2020.575828

**Published:** 2020-12-03

**Authors:** Barbara De Siena, Nicoletta Campolattano, Gianluca D’Abrosca, Luigi Russo, Daire Cantillon, Rosangela Marasco, Lidia Muscariello, Simon J. Waddell, Margherita Sacco

**Affiliations:** ^1^Dipartimento di Scienze e Tecnologie Ambientali Biologiche e Farmaceutiche, Università degli Studi della Campania Luigi Vanvitelli, Caserta, Italy; ^2^Department of Global Health and Infection, Brighton and Sussex Medical School, University of Sussex, Brighton, United Kingdom

**Keywords:** mycobacteria, efflux pump, antimicrobial drugs, multi drug resistance, biofilm, transmembrane protein, 3D structure modeling

## Abstract

Multi-drug resistant tuberculosis (MDR-TB) represents a major health problem worldwide. Drug efflux and the activity of efflux transporters likely play important roles in the development of drug-tolerant and drug-resistant mycobacterial phenotypes. This study is focused on the action of a mycobacterial efflux pump as a mechanism of drug resistance. Previous studies demonstrated up-regulation of the TetR-like transcriptional regulator *MSMEG_3765* in *Mycobacterium smegmatis* and its ortholog *Rv1685c* in *Mycobacterium tuberculosis* (*Mtb*) in acid-nitrosative stress conditions. MSMEG-3765 regulates the expression of the *MSMEG_3762/63/65* operon, and of the orthologous region in *Mtb* (*Rv1687c/86c/85c*). MSMEG-3762 and Rv1687c are annotated as ATP-binding proteins, while MSMEG-3763 and Rv1686c are annotated as trans-membrane polypeptides, defining an ABC efflux pump in both *M. smegmatis* and *Mtb*. The two putative efflux systems share a high percentage of identity. To examine the role of the putative efflux system MSMEG-3762/63, we constructed and characterized a *MSMEG-3763* deletion mutant in *M. smegmatis* (∆*MSMEG_3763*). By comparative analysis of wild type, knockout, and complemented strains, together with structural modeling and molecular docking bioinformatics analyses of the MSMEG-3763 trans-membrane protein, we define the protein complex MSMEG-3762/63 as an efflux pump. Moreover, we demonstrate involvement of this pump in biofilm development and in the extrusion of rifampicin and ciprofloxacin (CIP), antimicrobial drugs used in first- and second-line anti-TB therapies.

## Introduction

Tuberculosis (TB) is still considered a public health alert worldwide, with 10 million people falling ill and an estimated 1.4 million deaths in 2018 (Global Tuberculosis Report 2019). The complexity of the disease is due to many factors including the ability of *Mycobacterium tuberculosis* (*Mtb*), the causative agent of TB, to enter slow/non-replicating states, to remodel metabolic and respiratory systems, to cause latent infection, together with the increasing emergence of drug-resistant *Mtb* (Ayabina et al., 2016; Dheda et al., 2017; Bekele et al., 2018; Gordon and Parish, 2018; Singh et al., 2019). The impact of TB is also worsened by the high rate of *Mtb* infections among patients with chronic diseases like diabetes mellitus and HIV infection (Hayashi and Chandramohan, 2018; Fernandez et al., 2020). Among people living with HIV, the progression from TB infection to disease occurs at a high rate, positioning TB as the leading cause of mortality in this population (Peters et al., 2019). Other factors are associated with more severe TB, like renal disease, air pollution, tobacco consumption, alcohol abuse, and an aging population (Hameed et al., 2018). In this scenario, multi-drug resistance (MDR-TB) represents a significant risk to global public health, and is considered one of the most urgent problems to address (Ayabina et al., 2016; Dheda et al., 2017; Singh et al., 2019). Alternative strategies to combat MDR disease are underway, like the development of sensitive tests for early diagnosis of drug-resistant TB, or identification of bacterial phenotypes through drug therapy as biomarkers of treatment success, or therapeutic targeting of host immune responses (Honeyborne et al., 2016; Wilder et al., 2018; Thapa et al., 2019; Ahmed et al., 2020; Tsenova and Singhal, 2020). Studies on the molecular mechanisms involved in the acquisition of drug-resistant phenotypes in mycobacteria also represent an important strategy to combat MDR disease (Hameed et al., 2018; Van Camp et al., 2020). In *Mtb*, drug resistance evolves through chromosomal mutations that impact drug degradation and modification or target alteration, which operate alongside intrinsic factors such as low cell wall permeability and an extensive efflux network (Singh et al., 2019). Drug extrusion mediated by efflux pumps induces drug tolerance that reduces drug efficacy and may play a pivotal role in the development of resistance to anti-TB drugs (Te Brake et al., 2018). Efflux pumps may also be important for bacterial survival within macrophages, by detoxifying intracellular bacilli from acidification, toxic metal accumulation and the effects of nitrogen and oxygen reactive intermediates (Ehrt and Schnappinger, 2009).

Although the direct study of *Mtb* is necessary to understand pathogenic mechanisms, model systems represented by fast-growing non-pathogenic *Mycobacteria*, including *Mycobacterium smegmatis*, have been used widely to highlight relevant aspects of *Mtb* physiology (Trauner et al., 2012; Agrawal et al., 2015). Previously, we characterized the regulatory system of two ortholog operons, *Rv1687/86/85c* in *Mtb* and *MSMEG_3762/63/65* in *M. smegmatis*, both annotated as ABC efflux pump systems. Cossu et al. (2013) showed that *Rv1685c* and *MSMEG_3765*, coding for TetR-like regulators, were upregulated in acid-nitrosative stress conditions, mimicking a macrophage-like intracellular environment. By GFP promoter probe and transcriptional analyses, we strengthened that observation and defined the TetR regulons. We also showed, by EMSA analysis, that the recombinant purified TetR MSMEG-3765 protein is able to bind both *MSMEG_3762* and *Rv1687c* promoters, indicating binding affinity of the *M. smegmatis* protein for the *Mtb* regulatory region (Perrone et al., 2017). This, together with the high percentage of identity between the two operons, suggests that the two efflux systems may work in an analogous way in *Mtb* and *M. smegmatis*. Based on these considerations and the potential importance of efflux in antimicrobial drug efficacy in *Mtb*, we explored the role of the *M. smegmatis* MSMEG-3762/63 efflux pump. Using genetic manipulation, structural modeling, and molecular docking of MSMEG-3763, we show that MSMEG-3762/63 is an efflux pump involved in the extrusion of first- and second-line anti-TB drugs, as well as playing a role in the development of mycobacterial biofilms.

## Materials and Methods

### Bacterial Strains and Culture Conditions

*Escherichia coli* TOP10 and DH5α were used for cloning. *M. smegmatis* mc^2^155 was used throughout this work. The *E. coli* strains were grown in Luria-Bertani (LB) broth, while *M. smegmatis* was cultured in Middlebrook 7H9 broth (Difco) containing 10% oleic acid-albumin-dextrose-catalase supplement (Becton Dickinson) and 0.05% Tween 80. All strains were grown at 37°C with shaking at 200 rpm. Hygromycin (200 μg ml^−1^ for *E. coli* and 100 μg ml^−1^ for *M. smegmatis*), kanamycin (50 μg ml^−1^ for *E. coli* and 25 μg ml^−1^ for *M. smegmatis*), 5-bromo-4-chloro-3-indolyl-β-d-galactopyranoside (Xgal 50 μg ml^−1^) and sucrose (2% w/v) were used for selection or screening as appropriate. Ciprofloxacin, Norfloxacin, and Ofloxacin stock solutions (10 mg/ml^−1^) were prepared in NaOH 1 M and Rifampicin stock solution (10 mg/ml^−1^) was prepared in methanol. Mycobacterial biofilms were grown in Sauton medium (0.5 g KH_2_PO_4_, 0.5 g MgSO_4_ 7H_2_O, 2.0 g citric acid, 0.05 g ferric ammonium citrate, 60 ml glycerol, 4.0 g asparagine, 0.1 ml ZnSO_4_ 1% solution, distilled water up to 1,000 ml, and pH 7.4).

### Construction of the *Mycobacterium smegmatis* (*ΔMSMEG_3763*) Deletion Mutant

Primer sequences and plasmids are listed in Supplementary Tables S1, S2, respectively. The *M. smegmatis* (*ΔMSMEG_3763*) mutant strain, carrying an in frame deletion in *MSMEG_3763*, was isolated using a two-step homologous recombination strategy (Parish et al., 1999). A 806 bp fragment (up), containing the upstream flanking regions of *MSMEG_3763* was PCR-amplified from *M. smegmatis* mc^2^155 genomic DNA using the forward upMS3763f and reverse upMS3763r primers. The amplified fragment (up) was cloned into HindIII and KpnI restriction sites of p2NIL, yielding the pBD01 plasmid. A 825 bp fragment (dw), containing the downstream flanking regions of *MSMEG_3763*, was PCR-amplified from *M. smegmatis* mc^2^155 genomic DNA using the forward dwMS3763f and reverse dwMS3763r primers. The amplified fragment (dw) was cloned into KpnI and PacI restriction sites of pBD01, yielding the pBD02 plasmid. To obtain the suicide delivery vector pBD03, the pGOAL19 cassette was cloned into the PacI restriction site of the pBD02 plasmid. pBD03 was electroporated into *M. smegmatis* mc^2^155 and single crossover events (SCOs), positive in the Xgal screening and resistant to kanamycin and hygromycin, were verified by colony PCR. Among the positive SCOs, a single colony was streaked onto fresh media without selection, and incubated at 37°C for 5 days to allow the second recombination event to occur, before selection on plates containing sucrose and Xgal. The white sucrose-resistant colonies were screened for kanamycin and hygromycin sensitivity, and then analyzed by colony PCR to confirm that a second recombination event had occurred, yielding a deletion of 771 bp in the coding region of *MSMEG_3763*. One positive clone was chosen for further experiments and herein named *M. smegmatis* (*ΔMSMEG_3763*). The in-frame deletion event in *M. smegmatis* (*ΔMSMEG_3763*) was verified by sequencing.

### Construction of the *Mycobacterium smegmatis* (*ΔMSMEG_3763 pBD04*) Complemented Strain

To complement the gene-inactivating mutation in *M. smegmatis* (*ΔMSMEG_3763*), a DNA fragment containing the 770 bp coding sequence of *MSMEG_3763* (including start and stop codons) was PCR-amplified from *M. smegmatis* mc^2^155 with forward cMS3763f and reverse cMS3763Salr primers (Supplementary Table S2). The forward primer included an optimized Shine–Dalgarno sequence. The resulting 805 bp fragment was cloned into the EcoRI-SalI sites of pMV306hsp (Addgene#26155) to obtain pBD04, carrying the *MSMEG_3763* gene under the control of the *Mycobacterium bovis* BCG *hsp60* promoter. The recombinant plasmid pBD04 was verified by PCR and sequencing (Microgem Laboratory Research). pBD04 was electroporated into *M. smegmatis* (*ΔMSMEG_3763*) to generate the complemented *M. smegmatis* (*ΔMSMEG_3763 pBD04*) strain. Recombinant clones were selected using kanamycin, and the presence of pBD04 was verified by plasmid extraction followed by PCR analysis.

### Ethidium Bromide Efflux Pump Inhibition Assay

Efflux pump activity was determined using ethidium bromide (EtBr) as previously described (Rodrigues et al., 2013). *Mycobacterium smegmatis* strains were grown in 7H9/OADC medium at 37°C to an OD_600_ of 0.6 (corresponding to 10^8^ CFU ml^−1^), the pellet washed and resuspended in PBS supplemented with 0.05% Tween80 and EtBr solution to final concentration of 2 μg ml^−1^. Aliquots of 195.5 μl of the bacterial suspension were added to 96 well microtitre plate containing 4.5 μl of water or 4.5 μl of verapamil (VPL) to a final concentration of 250 μg ml^−1^. The assay was conducted at 37°C in a Promega GloMax® Discover Microplate Reader using the excitation and emission wavelengths of 520 nm and 560–640 nm, respectively. Fluorescence data was acquired every 60 s for 90 min at 37°C. Fluorescence values were expressed as percentage increase with respect to the value of each sample at 1 min. EtBr and VPL were used at ¼ the *M. smegmatis* minimum inhibitory concentration (MIC) in order not to compromise the cellular viability, as confirmed by c.f.u. counting. Each experiment was repeated in triplicate.

### MIC Determination

The MIC determination protocol was adapted from Agrawal et al. (2015). In brief, wells of a 96 well microtitre plate were filled with 50 μl 7H9 media except for the first column. Double of the required drug concentration was prepared and 100 μl volumes were added to the first column. This was diluted to reach the desired concentration of rifampicin, or serially diluted to halve the concentration by mixing with media only in the subsequent wells to the penultimate column for CIP. No drug was added to the last column as a no antibiotic control. The *M. smegmatis* strains were grown in replicates in 7H9 medium to an OD_600_ of 0.6, diluted 1,000 times (corresponding to 10^5^ CFU ml^−1^; Supplementary Figure S1), and 50 μl of the diluted culture was added to each well. The plate was sealed with parafilm to avoid drying of the cultures, and incubated with mild shaking (100 rpm) at 37°C for 40 h, followed by addition of 30 μl Sigma Resazurin sodium salt dye (filter sterilized, 0.2 mg ml^−1^ final concentration) to each well and incubation for a further 6 h before imaging. For EtBr and VPL MIC determination, 20 μl of Promega CellTiter-Blue® was used as indicator. MICs were determined as the values of the first well showing no growth as indicated by color change.

### Biofilm Formation and Quantification Assay

Biofilm formation assay was performed as previously described but with some modifications (O’Toole 2011). Bacterial cultures were grown in 7H9/ADC 0.05% Tween80 medium. The well-grown primary cultures (~OD_600_ 1.5) of wild-type, *M. smegmatis* (*ΔMSMEG_3763*) and *M. smegmatis* (*ΔMSMEG_3763 pBD04*) strains were washed twice with Sauton medium to remove Tween80. The cultures were diluted 1:500 to inoculate 7 ml Sauton medium in 60 mm diameter polystyrene Petri dishes. Petri dishes were incubated for 3–6 days at 37°C in a humidified incubator, and the formation of surface pellicles was monitored. Quantification of biofilm was performed as follows: well-grown primary cultures (~OD_600_ 1.5) were washed with Sauton medium and diluted to a final OD_600_ of 0.05 in the same medium, then distributed into 96-well polystyrene microtitre plates and incubated at 37°C in a humidified incubator for 4–7 days. Each well received an inoculum of 200 μl. After incubation, contents of the wells were aspirated out by syringe and the wells were washed with sterile water. The wells were then stained for 15 min, adding 300 μl 1% Crystal Violet (w/v) solution to each well. The stain was removed, washing the plate with sterile water, and plates were left to air dry. 300 μl 80% ethanol was added to each stained well and the plates were incubated for 15 min at room temperature. The content of each well was mixed by pipetting, and 200 μl transferred to an optically clear flat-bottom 96-well plate. Optical density (OD) was measured at 550 nm using a Biotek Synergy HT plate reader.

### 3D Structure Modeling

Amino acid sequences were retrieved and analyzed with ExPASy tools using the *MSMEG_3763* gene sequence as input. The MSMEG-3763 aggregation profile was evaluated using the AGGRESCAN server and the presence of putative transmembrane helices predicted by the TMHMM server on the basis of the amino acid sequence (Krogh et al., 2001). The three-dimensional (3D) structure of MSMEG-3763 was predicted using the I-TASSER server (Interactive Threading ASSEmbly Refinement; Roy et al., 2010). I-TASSER is a computational structure modeling method that uses a combinatorial approach of comparative modeling, threading, and *ab initio* modeling (Roy et al., 2010). In particular, the structure predictions by I-TASSER rely on template proteins with known structures obtained from databases and the prediction procedures are based on matching the query sequence against a non-redundant sequence database. According to I-TASSER criteria, the selection of the representative 3D model was performed by evaluating the C-score. This latter parameter is a scoring function based on the relative clustering structural density and the consensus significance score of the multiple threading templates. The C-score is usually in the range from −5 to 2, where a score of higher value indicates a predicted model with a high confidence and vice versa. Additionally, the quality of the selected model was further evaluated using PROCHECK and MolProbity (Laskowski et al., 1996; Davis et al., 2007). The selected 3D model was visualized using PyMol (The PyMOL Molecular Graphics System, Version 2.0 Schrödinger, LLC) and Chimera (Pettersen et al., 2004). The secondary structure elements were identified by DSSP (Hess et al., 2008; Joosten et al., 2011; Huang and MacKerell, 2013). The membrane protein modeling of MSMEG-3763 was performed using a two-step procedure. First, the protein was dissolved in dipalmitoyl phosphatidylcholine (DPPC) phospholipid bilayer by GROMACS (Hess et al., 2008) using the CHARMM36 force field (Huang and MacKerell, 2013). The protein was inserted into the membrane using the structural information obtained as reported above by TMHMM server. Finally, in order to define the correct orientation and position of MSMEG-3763 inside the membrane a minimization protocol was applied.

### Molecular Docking Studies

Molecular docking studies of rifampicin and ciprofloxacin with MSMEG-3763 were conducted using EAdock algorithm implemented in the Swissdock server (Grosdidier et al., 2011). The molecular docking protocol involved the preparation of MSMEG-3763 and ligand structures. In particular, for the protein the predicted 3D model was used as the reference structure, whereas the rifampicin and ciprofloxacin structures were downloaded from Protein Data Bank (PDB) database. In accord with the docking protocol, all residues of the protein were held fixed and a binding pocket was not defined. The docking procedure was validated by comparing the binding affinity values estimated for the ciprofloxacin/MSMEG-3763 (-5.9 kcal/mol) and rifampicin/MSMEG-3763 (-6.1 kcal/mol) complexes with that obtained from the ofloxacin/MSMEG-3763 (-4.2 kcal/mol) model. The binding affinity values were obtained for each docking model by using PRODIGY webserver.^1^ All docked poses were visualized and analyzed using Chimera (Pettersen et al., 2004).

## Results

### Deletion of *MSMEG_3763* Reduces the Ability of *Mycobacterium smegmatis* to Efflux Etbr

The *M. smegmatis* (*ΔMSMEG_3763*) mutant strain, carrying a 771 bp deletion in the coding region of *MSMEG_3763*, and its isogenic *M. smegmatis* (*ΔMSMEG_3763 pBD04*) complemented strain were constructed to characterize the function of the putative MSMEG-3762/63 mycobacterial efflux pump. The growth rate of the mutant and complemented strains were comparable to that of wild type in standard laboratory conditions (data not shown).

**Figure 1:**
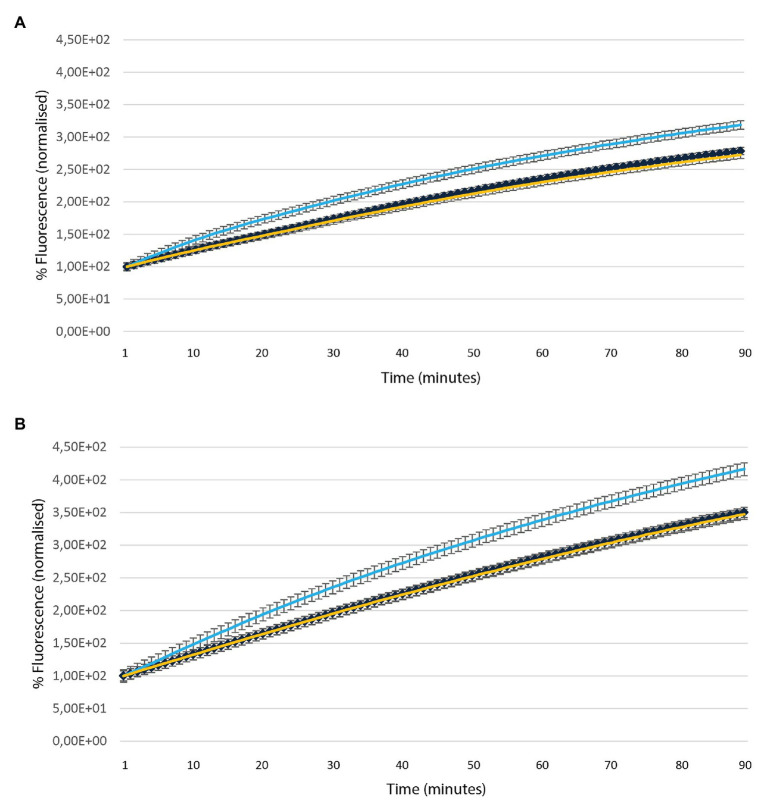
Accumulation of ethidium bromide (EtBr) over time in the absence **(A)** and presence **(B)** of verapamil (VPL) in *Mycobacterium smegmatis* (wt – yellow), deletion (*Δ*MSMEG_3763 – light blue) and complemented (ΔMSMEG_3763 pBD04 – dark blue) strains. The assay was conducted at 37°C. All the experiments were performed three times in triplicate.

Ethidium Bromide accumulation is a well-established method to monitor efflux activities in bacteria. The assay was used to assess the role of MSMEG-3763 as a component of the putative efflux pump by measuring differences in EtBr accumulation between wild type *M. smegmatis* (wt), deletion mutant (*ΔMSMEG_3763*), and complemented (*ΔMSMEG_3763 pBD04*) strains in the absence and presence of VPL, an efflux pump inhibitor (EPI). First, MICs of EtBr and VPL were determined as 8 μg ml^−1^ and 900 μg ml^−1^ for EtBr and VPL, respectively (data not shown). The efflux inhibition assays were conducted using concentrations that did not affect mycobacterial viability (¼ MICs). As expected, the *M. smegmatis* (*ΔMSMEG_3763*) mutant strain showed higher EtBr accumulation compared to the wild type and the complemented strains (Figure 1A). When the bacterial cultures were exposed to both EtBr and VPL, increased EtBr accumulation was observed for the three strains over the same incubation time. Once again, EtBr accumulation was higher for the mutant compared to the wild type and the complemented strains (Figure 1B), demonstrating that loss of MSMEG-3763 reduced the ability of mycobacteria to remove EtBr, and suggesting that this complex possessed efflux pump activity.

### Deletion of *MSMEG_3763* Increases the Sensitivity of *Mycobacterium smegmatis* to Antimicrobial Drugs

**Figure 2:**
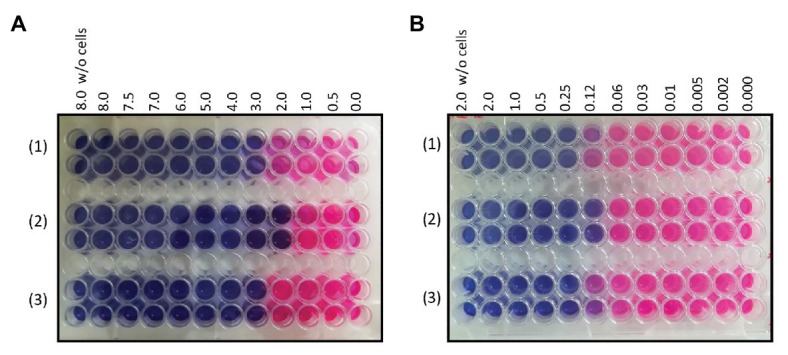
**(A)** Minimum inhibitory concentration (MIC) determination of rifampicin for (1) *M. smegmatis* wt (3 μg ml^-1^), (2) *M. smegmatis* (ΔMSMEG_3763; 2 μg ml^-1^), and (3) *M. smegmatis* (ΔMSMEG_3763 pBD04; 3 μg ml^-1^); **(B)** MIC determination of ciprofloxacin (CIP) for *M. smegmatis* wt (0.25 μg ml^-1^), *M. smegmatis* (ΔMSMEG_3763; 0.12 μg ml^-1^), and *M. smegmatis* (ΔMSMEG_3763 pBD04; 0.25 μg ml^-1^). MICs were determined by Resazurin Reduction Microtiter Assays (REMA). All experiments were performed three times in triplicate.

In order to verify the involvement of the MSMEG-3762/63 efflux pump in the removal of antimicrobial compounds used in anti-TB therapy, the MICs for first- and second-line anti-TB drugs were determined using the Resazurin Reduction Microtiter Assay (REMA). The MIC of rifampicin was lower (2 μg ml^−1^) for *M. smegmatis* (*ΔMSMEG_3763*) compared to M. smegmatis (wt; 3 μg ml^−1^; Figure 2A). As expected, the MIC of rifampicin for complemented *M. smegmatis* (*ΔMSMEG_3763 pBD04*) was the same as that observed for the wild type strain. The loss of MSMEG-3763 did not change the efficacy of other first-line antibiotics, isoniazid, and pyrazinamide (data not shown). The MICs of second-line TB antibiotics were also determined. An increase in sensitivity of *M. smegmatis* (*ΔMSMEG_3763*) to ciprofloxacin was observed. The MICs of ciprofloxacin for *M. smegmatis* (wt) and complemented *M. smegmatis* (*ΔMSMEG_3763 pBD04*) were (0.25 μg ml^−1^), while the MIC for *M. smegmatis* (*ΔMSMEG_3763*) was 0.12 μg/ml^−1^(Figure 2B). No difference in MIC was observed for other second-line anti-TB drugs, NOR or ofloxacin (data not shown). Thus, abrogation of MSMEG-3762/63 efflux pump function increased mycobacterial drug sensitivity to both rifampicin and ciprofloxacin.

### MSMEG-3763 Contains Predicted Transmembrane Helices

**Figure 3:**
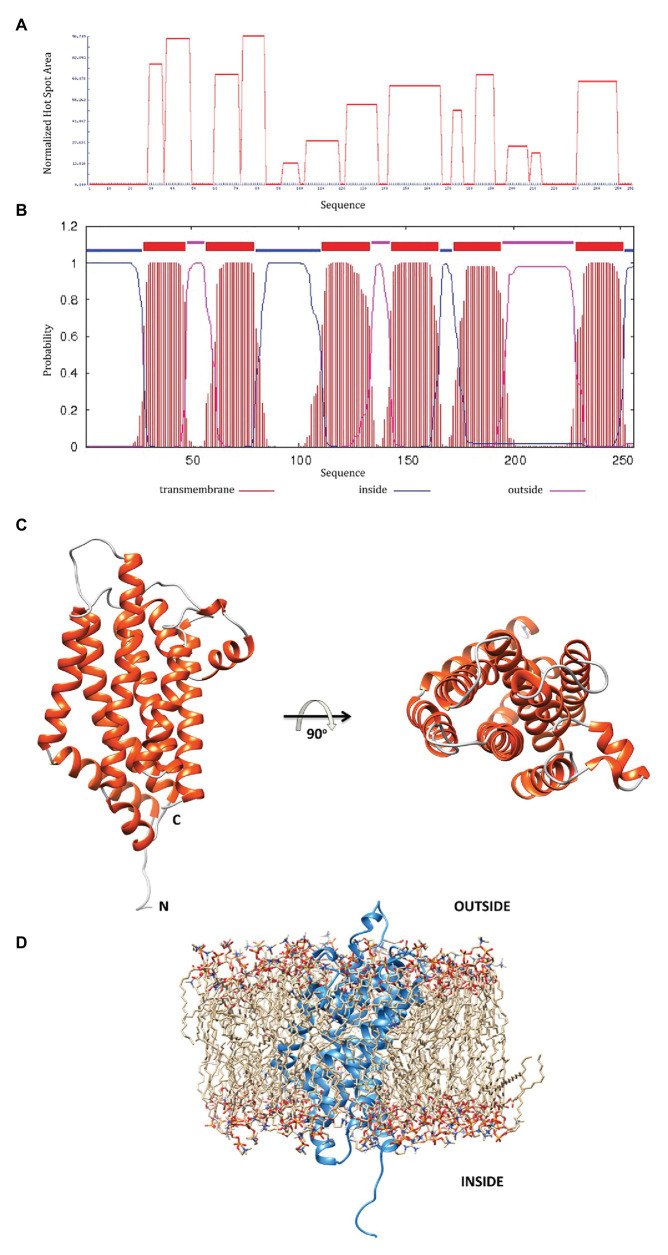
**(A)** The MSMEG-3763 protein aggregation profile using the AGGRESCAN server; **(B)** prediction of transmembrane helices by the TMHMM server; **(C)** MSMEG-3763 protein 3D model structure, calculated using the I-TASSER algorithm, where the six α-helices that likely form a channel inside the membrane are shown in red; **(D)** A proposed model placing MSMEG-3763 in the lipid double layer of the cytosolic membrane, according to TMHMM server modeling.

A bioinformatic analysis of the MSMEG-3763 protein based on its primary aa sequence was performed. The aggregation profile of the protein was determined using AGGRESCAN and TMHMM servers (Figure 3A), showing that the regions spanning residues 29–35, 37–48, 60–71, 73–83, 121–135, 141–165, 182–190, and 230–248 are clearly characterized by high aggregation prone sequences. Further analysis using the server TMHMM suggested that, in the regions showing high aggregation propensity, the protein may form transmembrane helices (Figure 3B).

### 3D Modeling of MSMEG-3763 Reveals a Channel Forming Structure

To better understand the functional proprieties of MSMEG-3763 a detailed description of the structural features is required. Therefore, to elucidate the molecular details of the protein, we performed 3D modeling of the structure using I-TASSER. The computational modeling of MSMEG-3763 generated five models with C-scores ranging from −4.36 to 0.29. The 3D model with the highest c-score was used as a reference for the structural analysis. Additionally, the good quality of the selected 3D model was confirmed by evaluating the structural statistics (Supplementary Figure S2A) and the Ramachandran plots (Supplementary Figure S2B) respectively, obtained by analyzing the predicted model using the software MolProbity (Davis et al., 2007), with 86 and 11% of aa-residues situated in most favorable (MF) and allowed regions (AR), and PROCHECK (Laskowski et al., 1996), with 84 and 13% aa-residues situated in MF and AR, respectively. The 3D structural model of MSMEG-3763 (Figure 3C) indicates that the protein presents as a globular fold composed of 10 α-helices (A = 10–27, B = 29–47, C = 60–91, D = 94–101, E = 106–136, F = 143–169, G = 172–188, H = 201–207, I = 213–223, and L = 232–257) joined by short non-helical regions that provide a rigid structural framework. In particular, the hydrophobic portion of the protein is a typical helix bundle composed of six α-helices (Figure 3C, in red) that likely forms a channel inside the membrane. As proposed by the model reported in Figure 3D, the hydrophilic portions, according to TMHMM predictions, probably correspond to the outer and the cytosolic regions of the protein. Therefore, structural modeling of the putative transmembrane component of the MSMEG-3762/63 efflux pump predicts a structure suited to purpose.

### Molecular Docking Analysis Suggests That Rifampicin and Ciprofloxacin Are Ligands of MSMEG-3763

**Figure 4:**
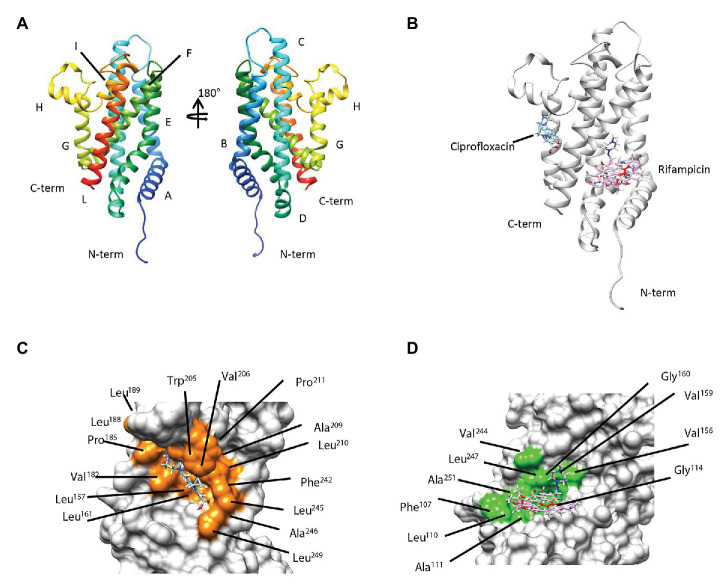
**(A)** Secondary and tertiary organization of MSMEG-3763 as proposed by the predicted 3D model. **(B)** Overlay of the three-dimensional (3D) models predicted by molecular docking studies of the protein-ligand complexes of MSMEG-3763 with ciprofloxacin (cyan) and rifampicin (magenta). Mapping of the residues of MSMEG-3763 involved in the interaction with ciprofloxacin (orange; **C**) and rifampicin (light green; **D**).

To gain structural insights into the interaction between rifampicin and ciprofloxacin compounds and the membrane protein MSMEG-3763 (Figure 4A), we performed a series of molecular docking studies that represent a powerful technique for studying protein-ligand complexes. In particular, molecular docking aims to predict at atomic level the binding mode (i.e., position and orientation) and affinity of a complex formed by two or more constituent molecules with known structures. The three-dimensional model of protein-ligand complexes of MSMEG-3763 was generated using the EAdock algorithm (Figure 4B). The optimized structure of the ciprofloxacin/MSMEG-3763 complex indicated that the recognition mechanism of ciprofloxacin by MSMEG-3763 is principally mediated by the residues Phe^107^, Leu^157^, Leu^161^, Val^182^, Pro^185^, Leu^188^, Leu^189^, Trp^205^, Val^206^, Ala^209^, Leu^210^, Pro^211^, Phe^242^, Leu^245^, Ala^246^, Leu^249^ forming a shallow hydrophobic pocket on the surface of MSMEG-3763 (Figure 4C). Instead, for the rifampicin/MSMEG-3763 complex (Figure 4D) the molecular docking structure predicted that rifampicin binds into a hydrophobic cleft on the surface of MSMEG-3763 formed by Phe^107^, Leu^110^, Ala^111^, Gly^114^, Val^156^, Val^159^, Gly^160^, Val^244^, Leu^247^, and Ala^251^. These docking results therefore suggest that this efflux pump has the capacity to bind, and potentially export, the antimicrobial drugs rifampicin and ciprofloxacin. To validate the docking protocol applied to obtain the structural model of ciprofloxacin/MSMEG-3763 and rifampicin/MSMEG-3763 complexes, we estimated for both models the binding affinity and then compared these values with that estimated for the ofloxacin/MSMEG-3763 model (Supplementary Figure S3) obtained using the same docking parameters. Notably, no difference in MIC of ofloxacin was observed for *M. smegmatis* wt and *∆3763* mutant strain. The comparison of the binding affinity values (ciprofloxacin/MSMEG-3763 = -5.9 kcal/mol; rifampicin/MSMEG-3763 = -6.1 kcal/mol; and ofloxacin/MSMEG-3763 = -4.2 kcal/mol) clearly indicates that the formation of ciprofloxacin/MSMEG-3763 and rifampicin/MSMEG-3763 complexes is favored with respect to the ofloxacin/MSMEG-3763 complex, confirming the MIC data.

These findings demonstrate that the applied docking protocol allows a structural model for ciprofloxacin and rifampicin binding to be obtained, providing a reasonable description of the molecular determinants involved in the recognition mechanism of MSMEG-3763 for both compounds.

### Inactivation of MSMEG-3763 Inhibits Biofilm Formation

**Figure 5:**
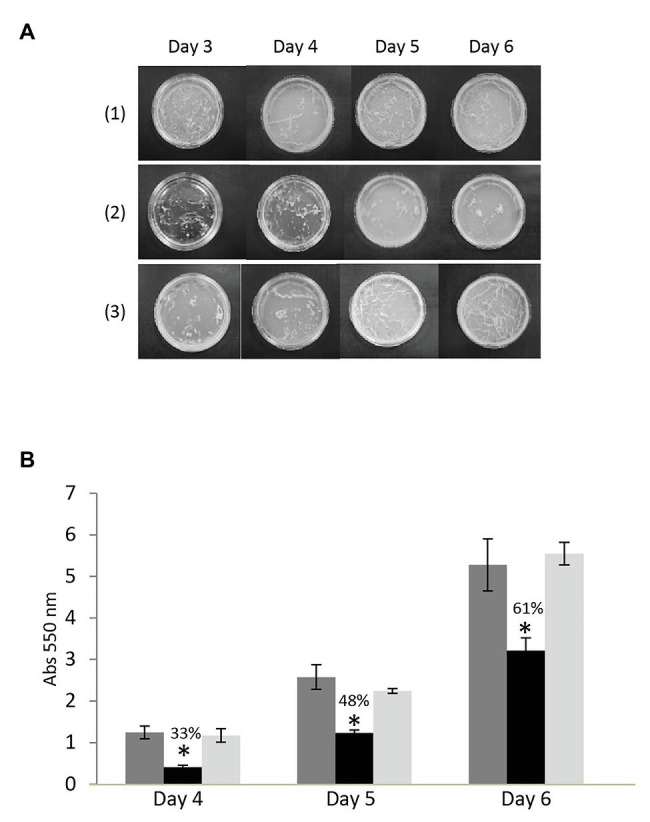
**(A)** A top-down view of mycobacterial pellicle biofilms after 3–6 days growth in detergent-free Sauton medium with (1) *M. smegmatis* wild type, (2) deletion (ΔMSMEG_3763), and (3) complemented (ΔMSMEG_3763 pBD04) strains. In contrast to the wild type, the *M. smegmatis* (ΔMSMEG_3763) mutant strain formed an untextured biofilm; normal pellicle formation was restored upon complementation. All the experiments were performed three times in triplicate; **(B)** Crystal violet quantification of biofilms of *M. smegmatis* wild type (wt – dark gray), deletion (ΔMSMEG_3763 – black), and complemented (ΔMSMEG_3763 pBD04 – light gray) strains. The biofilm mass formed at days 4, 5, and 6 was quantified spectrophotometrically at 550 nm after crystal violet staining. All the experiments were performed three times in triplicate. Error bars indicate the standard deviation of the mean values. ^*^p < 0.001.

Bacterial efflux pumps have roles in many physiological functions that influence pathogenicity besides drug export, including biofilm development. To test if the absence of MSMEG-3763 affected the ability to form a mycobacterial biofilm, a comparative analysis of the three strains was performed. Pellicles formed by *M. smegmatis* (wt) were observed on the surface after 3 days of incubation and continued to develop up to the last day of observation (day 6; Figure 5A). The *M. smegmatis* (*ΔMSMEG_3763*) deletion mutant also formed a biofilm, but the reticulated appearance associated with formation of the extracellular matrix was not observed on the surface of the liquid growth medium. This observation was further confirmed by a crystal violet quantification of the biofilms (Figure 5B). The amount of biofilm formed by the efflux pump mutant *M. smegmatis* (*ΔMSMEG_3763*) was reduced from 33–61%, depending on the day of sampling, compared with the wild type. As expected, the ability to form a biofilm was restored nearly to wild type levels in the *M. smegmatis* (*ΔMSMEG_3763 pBD04*) complemented strain (Figure 5B). Loss of efflux function through MSMEG-3762/63 therefore interferes with biofilm development, influencing mycobacterial interactions with the environment that may affect pathogenicity.

## Discussion

Multi-drug resistance of an increasing number of pathogens represents a growing health problem worldwide. This is particularly urgent for diseases like tuberculosis, mainly caused by *Mtb*, or nosocomial infections caused by opportunistic pathogens such as *Staphylococcus aureus*, *Pseudomonas aeruginosa*, and *Acinetobacter baumannii* (Dheda et al., 2017; Botelho et al., 2019; Guo et al., 2020; Moubareck and Halat, 2020). Antibiotic resistance may be conferred through different mechanisms, such as antibiotic degradation or modification, receptor alteration, or antibiotic efflux mediated by membrane transport systems (Machado et al., 2017; Singh et al., 2019; Van Camp et al., 2020). Efflux transporters are a serious problem to the efficacy of antimicrobial drugs as they confer to bacteria the capacity to rapidly export drugs and to evade current drug therapies (Alcalde-Rico et al., 2016; Du et al., 2018). For instance, in *Staphylococcus aureus*, over-expression of efflux pumps has been associated with high levels of resistance to many antibiotics (Hassanzadeh et al., 2020).

Although efflux pumps are mostly studied for their role in antibiotic resistance, there is growing evidence of their involvement in a variety of bacterial behaviors related to phenotypes associated with virulence, including biofilm development and quorum sensing-dependent expression of virulence factors (Piddock, 2006; Alcalde-Rico et al., 2016; Alav et al., 2018; Du et al., 2018). For instance, in *P. aeruginosa*, where a high percentage of virulence genes are under the control of different quorum sensing systems, the involvement of efflux pumps in extruding intercellular signal molecules or their precursors, as in the case of the MexAB, MexGHI, and MexEF efflux systems, has been reported, in addition to roles in biofilm development and secretion of host-produced antimicrobial compounds (Minagawa et al., 2012). Efflux pumps are also emerging as functionally-relevant in pathogen-host interactions. Pasqua et al. (2019) reported that the EmrKY efflux pump is required for survival of *Shigella* in the macrophage environment, highlighting for the first time the key role of an efflux pump during the *Shigella* invasive process. Furthermore, the ABC family efflux pump MacAB is required for survival of *Salmonella enterica* serovar Typhimurium inside the macrophage environment, where they are exposed to ROS; indeed, deletion mutants of *macAB* genes showed weakened intracellular replication within macrophages and an impaired growth in the inflamed intestine (Bogomolnaya et al., 2013; Alcalde-Rico et al., 2016).

In *Mtb*, *Rv1685c*, coding for a TetR-like protein, and its ortholog *MSMEG_3765* in *M. smegmatis* were found to be upregulated in acid-nitrosative stress conditions, mimicking the macrophage environment (Cossu et al., 2013). Members of the TetR family of transcriptional regulators are widespread among bacteria, especially in microorganisms exposed to environmental stresses, being involved in the control of many cellular functions, including efflux pump activity (Balhana et al., 2015). We have previously demonstrated that in *M. smegmatis*, the TetR-like protein MSMEG-3765 (TetR3765) regulates expression of its own operon, coding for two other proteins annotated as components of an ABC efflux pump, and that the recombinant purified TetR3765 protein is able to bind both *MSMEG_3762/63/65* and *Rv1687/86/85c* upstream sequences, indicating binding affinity of the *M. smegmatis* protein for the *Mtb* regulatory region (Perrone et al., 2017). These results suggested that the same regulatory system exists for expression of the putative MSMEG-3762/63 and Rv1687/86c efflux pumps, which share a high level of amino acid sequence identity.

Here, we used isogenic *M. smegmatis* deletion (*ΔMSMEG_3763*) and complemented *M. smegmatis* (*ΔMSMEG_3763* pBD04) strains to characterize the MSMEG-3762/63 efflux pump. The role of this gene cluster annotated as efflux pump components was confirmed by EtBr accumulation assay, where loss of the system resulted in faster accumulation of EtBr in the mycobacterial cell. A comparative analysis of wild type, deletion and complemented strains revealed that the MSMEG-3762/63 efflux system is involved in biofilm development, a phenotype associated to virulence and demonstrated to depend upon activity of efflux pumps in various pathogens (Alav et al., 2018).

Using structural modeling tools, we show that MSMEG-3763 contains several regions with high aggregation propensity that may form transmembrane helices. The predicted three-dimensional structure indicates that the protein presents as a globular fold composed of 10 α-helices. Moreover, the structural data demonstrated that the fold is further stabilized by hydrophobic interactions between residues of the six central α-helices that show a typical helix bundle topology, that likely has a crucial role in the formation of a trans-membrane channel.

Efflux systems contribute to the intrinsic resistance of bacteria to antimicrobial drugs (Singh et al., 2019). In *Mtb*, up-regulation of *Rv0194* and *mmpL5*, coding for efflux pump components, occurred in a higher number of rifampicin-resistant compared to rifampicin-susceptible clinical isolates, highlighting a potential role of these efflux systems in the development of drug resistance in patients (Narang et al., 2019). Therefore, we determined the sensitivity of *M. smegmatis* (*∆MSMEG_3763*) to anti-TB drugs, which indicated that the MSMEG-3762/63 complex is involved in the efflux of rifampicin and ciprofloxacin, first- and second-line anti-TB drugs, respectively. Based on these results, we mapped the structural details regulating the complex formation between MSMEG-3763 and the two drug ligands by computational docking. In the case of ciprofloxacin, the molecular docking studies reveal that complex formation is controlled by a shallow hydrophobic cleft formed by residues located in the α-helices E, F, G, H, and L; while for rifampicin the binding mechanism is suggested to be principally modulated by residues situated in the regions belonging and surrounding the E, F, and L α-helices.

Efflux pumps represent important therapeutic targets in the treatment of MDR infectious diseases, included TB, evidenced by recent inhibition of the essential mycobacterial efflux pump, EfpA (Johnson et al., 2019, 2020). Indeed, the possibility of EPIs as candidate molecules to treat TB is widely studied (Kumar et al., 2016). Rational drug design alongside a deeper understanding of efflux pump physiology and structure will lead to new more effective therapeutic strategies for MDR disease (Reza et al., 2019; Zarate et al., 2019).

## Data Availability Statement

The raw data supporting the conclusions of this article will be made available by the authors, without undue reservation.

## Author Contributions

BS, NC, and RM performed the experimental work under the supervision of LM and MS, and BS and DC performed EtBr accumulation assays and biofilm development assays under the supervision of SW, at the University of Sussex. GA and LR performed the bioinformatics work. BS, LR, and MS wrote the manuscript with a consistent collaboration of SW. All authors contributed to the article and approved the submitted version.

### Conflict of Interest

The authors declare that the research was conducted in the absence of any commercial or financial relationships that could be construed as a potential conflict of interest.

## Footnotes

^1^https://bianca.science.uu.nl/prodigy/
