# Supplementary material for: Characterization of the Mycobacterial MSMEG-3762/63 Efflux Pump in Mycobacterium smegmatis Drug Efflux
Source: Front Microbiol. 2020 Dec 3;11:575828. doi: 10.3389/fmicb.2020.575828 (PMC7744416; doi:10.3389/fmicb.2020.575828)
Supplement: Supplementary file 1 [file Data_Sheet_1.docx]

Supplementary Material

# Supplementary Tables

| **Primer** | | **Sequence** | **Description** |
| --- | --- | --- | --- |
| **Construction of the *M. smegmatis ΔMSMEG_3763* mutant strain** | | | |
| upMS3763f | | 5’-CCCAAGCTTCCGGACGACGGCAGGCCTAG-3’ | Forward primer for the amplification of the upstream *MSMEG_3763* fragment |
| upMS3763r | | 5’-GGGGTACCCCGCCGAACACTCACGCTGCC-3’ | Reverse primer for the amplification of the upstream *MSMEG_3763* fragment |
| dwMS3763f | | 5’-GGGGTACCCTGCGCAGACGGACACCATGA-3’ | Forward primer for the amplification of the downstream *MSMEG_3763* fragment |
| dwMS3763r | | 5’-CCTTAATTAACTGGTCTGCCCGCAGGATCGCG-3’ | Reverse primer for the amplification of the downstream *MSMEG_3763* fragment |
| **Construction of the *M. smegmatis ΔMSMEG_3763* complemented strain** | | | |
| cMS3763f | 5’-CGGAATTCAGAAGGAGAAGTACCGATGAGTGTTCGGCGG-3’ | | Forward primer for the amplification of *MSMEG_3763* gene |
| cMS3763Salr | 5’-ACGCGTCGACTCATGGTGTCCGTCTGCGCAG-3’ | | Reverse primer for the amplification of *MSMEG_3763* gene |

**Supplementary Table 1.** Oligos used in this work.

| **Name** | **Description** | **Reference** |
| --- | --- | --- |
| **Construction of the *M. smegmatis ΔMSMEG_3763* mutant strain** | | |
| p2NIL | High copy suicide vector with Kanamycin resistance | Parish et al., 1999 |
| pBD01 | p2NIL+*MSMEG_3763* upstream fragment | This work |
| pBD02 | pBD01+*MSMEG_3763* downstream fragment | This work |
| pGOAL19 | Marker Cassette conferring ampicillin resistance, sucrose sensibility and carrying the LacZ gene. | Parish et al.,1999 |
| pBD03 | pBD02+pGOAL19 marker cassette | This work |
| **Construction of the *M. smegmatis ΔMSMEG_3763* complemented strain** | | |
| pMV306hsp | Mycobacteria integrating vector with hsp60 promoter | Andreu et al., 2010 |
| pBD04 | pMV306hsp+MSMEG_3763 coding sequence | This work |

**Supplementary Table 2.** Plasmids used in this work.

# Supplementary Figures


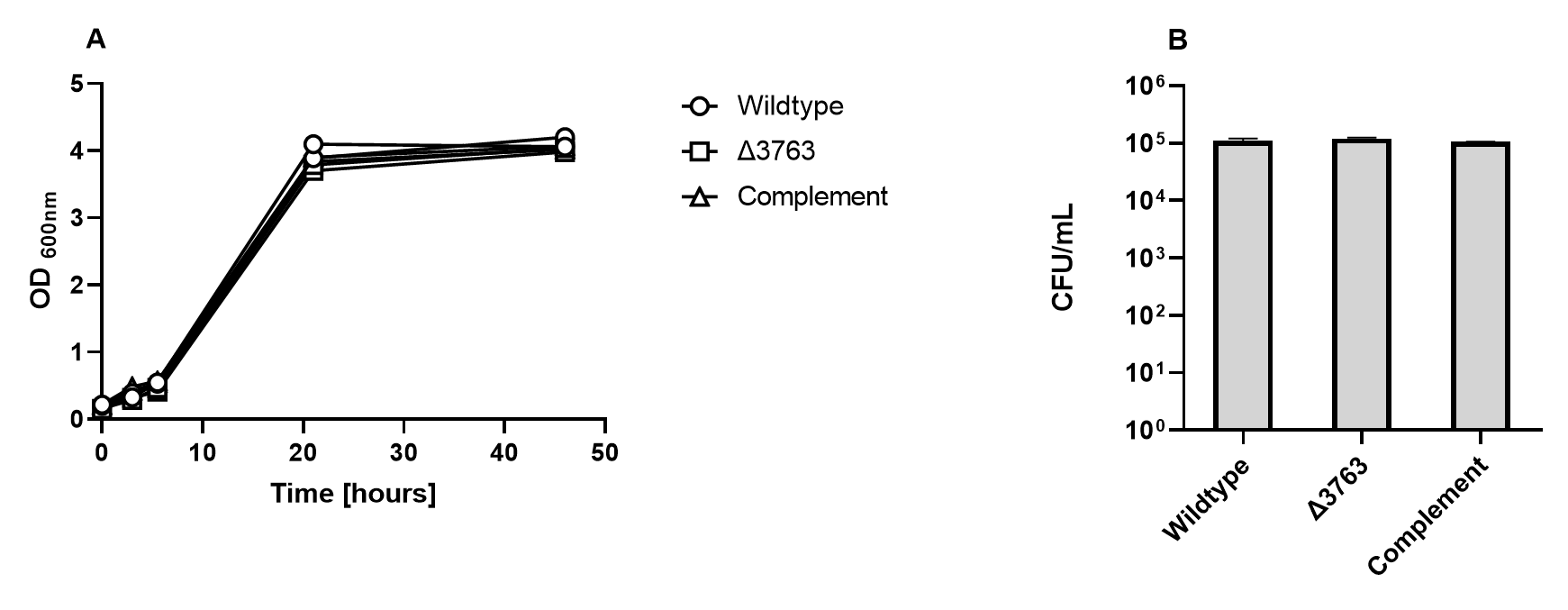


**Supplementary Figure 1.** (A) *M. smegmatis* cultures were grown to log phase (OD_600_ = 0.6) and diluted to 0.0006 for plating, corresponding to 1 x 10^5^ CFU/mL. (B) Data points are the mean of two pooled experiments ± standard deviation.


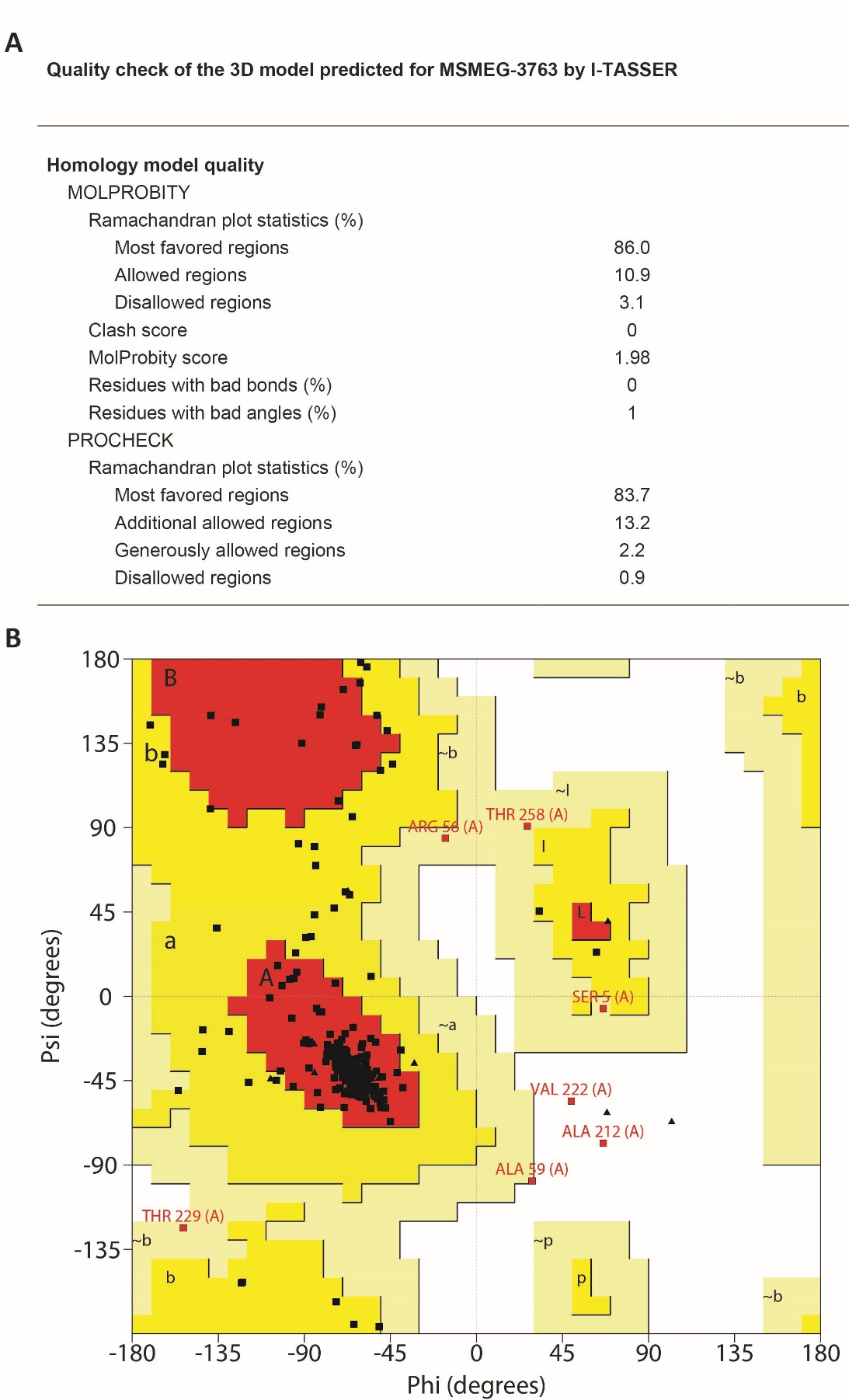


**Supplementary Figure 2.** (A) Quality check of the 3D structural model predicted for MSMEG-3763 by using I-TASSER. (B) Ramachandran plot carried out by PROCHECK.


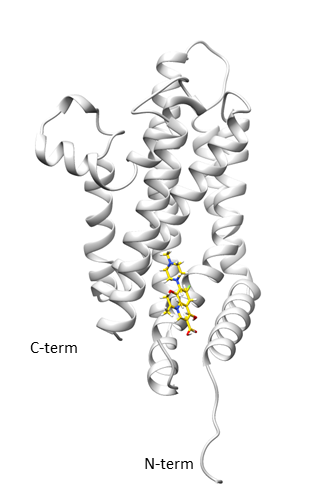


**Supplementary Figure 3.** Structural model obtained for the ofloxacin/MSMEG-3763 complex by using the EAdock algorithm implemented in the Swissdock server. The ofloxacin molecule is reported as yellow stick.
